# Evaluation of some immunological markers in co-infection of COVID-19 with thrush candidiasis

**DOI:** 10.1590/1806-9282.20230845

**Published:** 2024-05-13

**Authors:** Heam Qaid Mohammed Al-Kenani, Orass Madhi Shaheed

**Affiliations:** 1 University of Al-Qadisiyah, College of Medicine, Department of Medical Microbiology – Diwaniya, Iraq.

**Keywords:** Biomarkers, COVID-19, Candidiasis, Co-infection, IL-8, IL-10

## Abstract

**OBJECTIVE:**

COVID-19 infection poses significant risks, including life-threatening consequences and fungus synchronization, making it a significant concern. This study seeks to assess the effect of concurrent infection of COVID-19 with Thrush *Candida albicans* on the patient's health state by measuring the proportion of immune cells and certain interleukins such as IL-8, -10, -17, and -33.

**METHODS:**

The study involved 70 patients (30 patients with COVID-19, 17 patients with thrush candidiasis, and 23 patients with Thrush *Candida albicans*) and 50 healthy individuals as a control group. COVID-19 was identified using RT-PCR, while *C. albicans* were identified through culture media, biochemical testing, and oral swabs. Ruby equipment and ELISA kits were used for blood counts and interleukin detection.

**RESULTS:**

COVID-19, thrush candidiasis, and Thrush *Candida albicans* infections occur in a wide range of age groups (4–80 years), with no significant differences between sexes (p>0.05). Immunologically, our study found that Thrush *Candida albicans* patients had the highest rate of neutrophils (89.6%) and basophils (2.01%), while corona patients had the highest percentage of lymphocytes (70.12%) and eosinophils (7.11%), and patients with thrush candidiasis had the highest percentage of monocytes. Thrush *Candida albicans* patients showed increased IL-8 (56.7 pg/mL) and IL-17 (101.1 pg/mL) concentrations, with the greatest concentration of IL-33 (200.5 pg/mL) in COVID-19, and a decrease in the level of IL-10 in patient groups compared with controls.

**CONCLUSION:**

Patient groups showed increased neutrophils, lymphocytes, monocytes, and IL-8 levels, with a significant linear association between proinflammatory interleukins and these cells.

## INTRODUCTION

The coronavirus disease, which is known as SARS-CoV-2, is a threat to public health. It can cause severe illness and require hospitalization^1^. Individuals with weak immune systems or medical conditions are more susceptible to severe COVID-19 and respiratory distress syndrome^2^. Critically ill patients with ARDS admitted to the ICU and need artificial ventilation are more susceptible to nosocomial fungal infections, with COVID-19 patients experiencing bacterial and fungal co-infections^3^. The prevalence of diseases has increased due to increased disease transmission and pesticide use^4^.

COVID-19 patients face candidiasis due to compromised immune systems, zinc and iron deficiencies, and iatrogenic and nosocomial transmissions, which are not fully understood^5^. COVID-19-related mucormycosis is the most common contagious infection in COVID-19 patients, while candidiasis, a less concentrated parasitic disease, has emerged in countries such as India, Iran, China, and the United Kingdom^6^.

Serious COVID-19 can cause severe cytokine delivery, immune weakness, and mortality in established patient populations. While superinfections were initially uncommon, reports of optional contagious diseases as potential entanglements are increasing^7^. COVID-19 patients with acute respiratory distress syndrome (ARDS) face challenges in clinical training due to aspiratory Aspergillosis and candidiasis, but immunological tools can improve infection control^8^.

Thrush candidiasis, caused by *Candida albicans*, is a severe parasitic disease causing clinical issues and COVID-19-related complications. The exact pathophysiology of candidiasis in COVID-19 patients is not fully understood, and there is limited information on contagious co-contaminations. Deficient understanding and lack of preventive measures may lead to misdiagnosis and potentially destroy COVID-19 outcomes^9^. The meaning of the sole impacts of the safe reaction on parasitic contaminations needs further examination^10^. Accordingly, this study meant to decide the job of a few safe cells as neutrophils, lymphocytes, monocytes, eosinophils, and basophils in extra to certain interleukins such as IL-8, -10, -17, and -33 in co-disease of COVID-19 with thrush candidiasis (CTC).

## METHODS

### Study design and sample collection

Before the start of the research project, this study was approved by the ethical committee of the Faculty of Medicine, University of AL-Qadisiyah, and informed consent was obtained from all individuals, and permission was obtained from the Al-Diwaniaya Teaching Hospital/Quarantine unit. A case-control study was conducted with individuals recruited from the Al-Diwaniaya Teaching Hospital/Quarantine unit in the Al-Qadisiyah governorate. The study was carried out during the period from December 1, 2021 to the end of January 2022. The age ranged between 4 and 80 years. The study involved 70 patients (30 patients with COVID-19, 17 patients with thrush candidiasis, and 23 patients with CTC), and 50 healthy individuals as a control group. Assent was taken from all members while gathering the survey and tests.

### Immunological study

Absolute IL-8, IL-10, IL-17, and IL-33 ELISA units (Demeditec/Germany) were utilized for the quantitative assurance of complete IL-8, IL-10, IL-17, and IL-33 in human serum. The reagents readiness and examination technique were done by the producer's depiction, an enzyme-linked immunosorbent assay (ELISA). The plate had been pre-coated with human IL antibody. ILs present in the sample were added and they bound to antibodies coated on the wells. Then, biotinylated human IL antibody was added and it bound to ILs in the sample. Then, Streptavidin-HRP was added and it bound to the biotinylated IL antibody. After incubation, unbound Streptavidin-HRP was removed during washing. The substrate solution was then added, and a color was developed in proportion to the amount of human ILs. The reaction was terminated by the addition of an acidic stop solution, and the absorbance was measured at 450 nm.

A complete blood count (CBC) was performed using the RUBY framework, using EDTA tubes and natural testing. After 1–5 min, a complete count of neutrophils, lymphocytes, monocytes, eosinophils, and basophils was displayed on a PC screen, with each patient's name and number printed.

Isolation and identification of C. albicans on Sabouraud dextrose agar were performed using brooding at 37°C for 48 h, revealing cream, smooth, pale curved states with minimal separation. Germ-tube test: The test showed *C. albicans* hyphal outgrowths in horse serum, followed by a biochemical test. Direct microbial examination: Morphological highlights of *C. albicans* were identified using a smear, revealing non-pigmented septate hyphae with distinctive dichotomous fanning at 45°. COVID-19 identification: Positive RT-PCR SARS-CoV-2 nasopharyngeal swab test confirms COVID-19. To confirm the infection with SARS-COV2, especially in mild infection, the individuals underwent rapid tests for the detection of IgG and IgM Abs.

### Statistical analysis

Data were converted into the modern database, cleaned using range and legitimate methods, and analyzed using expert guidance for factual examinations using SPSS 20 and Microsoft Excel 2010.

## RESULTS

**Figure 1:**
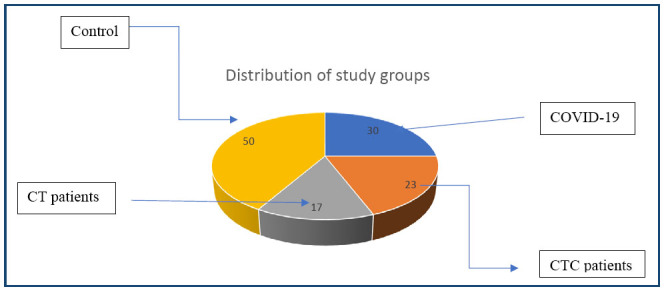
Frequency distribution of study groups.

Demographic characteristics of patients and controls are shown in Table 1 and Figure 1. The ages of patients varied from 4 to 80 years, with an average age of 36.11, 38.7, and 40.92 years for COVID-19, CTC, and TC patients, respectively. Results found no significant differences in age or gender distribution in fungi or virus infection rates between groups.

**Table 1:** Demographic characteristics of patients and controls.

Age statistic	COVID-19 patients	CTC patients	CT patients	Control	p-value
Age range (years)	4–80	4–80	4–80	4–80 years	
Mean	36.11	38.7	40.92	38.3	0.155
SD	9.85	11.61	13.4	11.77	
SE	1.80	2.42	3.25	1.66	
Number	30	23	17	50	
Females	17	11	10	23	0.085
Males	13	12	7	27	0.052

CTC: COVID-19 and thrush candidiasis; CT: thrush candidiasis.

Total blood cell count shows immune cells increased in COVID-19 patients, with some increasing in both the virus and fungus simultaneously. Neutrophils and lymphocytes were highest in COVID-19 patients, followed by CTC and TC patients.

Individuals with TC and CTC had the highest percentages of monocytes (25.69 and 23.67%), while patients with COVID-19 and CTC had the largest percentages of eosinophils (7.11%) and basophils (2.01%). Significant differences were observed in immune cell proportions compared with healthy individuals (Table 2).

**Table 2:** Evaluation of immune cells in studied groups.

Immune cells count	COVID-19 patients	CTC patients	CT patients	Control	X^2^	p-value
Neutrophils %	85.3*	89.6*	77.9*	39.44	1.071	0.089
Lymphocytes %	70.12*	67.3"	67.26*	19.32	0.853	0.100
Monocytes %	20.75*	23.67*	25.69"	4.99	0.555	0.114
Eosinophils %	7.11*	3.61*	1.68*	0.76	0.304	0.261
Basophils %	1.66*	2.01*	0.77*	0.27	0.300	0.329

* Significant differences in comparison with control (p<0.05);"nonsignificant differences in comparison with control (p<0.114); X^2^: chi square; CTC: COVID-19 and thrush candidiasis; CT: thrush candidiasis.

The study found increased proinflammatory interleukins IL-8, -17, and -33 in patient groups compared with healthy controls. Anti-inflammatory IL-10 concentrations were low in patients but not statistically significant. IL-8 and IL-17 concentrations were highest in CTC patients, while IL-33 was higher in COVID-19 and CTC. The lowest levels of IL-10 were found in CT and CTC serum (Table 3).

**Table 3:** Evaluation of some interleukins in studied groups.

Interleukins	COVID-19 patients	CTC patients	CT patients	Control	X^2^	p-value
IL-8 (pg/mL)	25.3*	56.7*	45.6*	3.17	8.07	0.041
IL-10 (ng/L)	4.31	2.81	2.44	4.73	0.52	0.093
IL-17 (pg/mL)	98.91*	101.1*	52.34*	9.61	9.84	0.029
IL-33 (pg/mL)	200.5*	193.5*	88.76*	10.31	17.11	0.002

* Significant differences in comparison with control (p<0.05); X^2^: chi square; CTC: COVID-19 and thrush candidiasis; CT: thrush candidiasis.

The study found a strong positive relationship between proinflammatory interleukins (IL-8, -17, and -33) and immune cells, particularly neutrophils and lymphocytes, in patients with viral and fungal infections. The relationship was weakly positive in fungal infections. High IL-33 concentrations are significantly linked to lymphocytes and neutrophils, as well as IL-17 and IL-8.

## DISCUSSION

*Candida albicans* co-infections with COVID-19 can occur in people of all ages, and they are associated with higher levels of immune cells and proinflammatory interleukins. Also, COVID-19 can cause immunosuppression or cytokine release, which can increase the risk of fungal infections with increased IL-18, IL-17, IL-33, neutrophils, monocytes, and basophils. According to Song et al.'s^11^ research, severe COVID-19 patients with better access to antibiotics, nutrition, and tests, as well as persistent neutropenia, are more likely to contract *Candida* species infections. The results of this study agree with that of Remy et al.^10^ who found that COVID-19 often causes immunological reactions affecting innate and adaptive immune responses, impacting clinical course and prognosis. Severe COVID-19 may cause immunosuppression or cytokine release, leading to morbidity and death, especially in elderly patients. COVID-19 increases the risk of fungal infections due to immune system influence and potential reduction in defenses from COVID-19 therapies^12^. The exact mechanism by which COVID-19 increases the risk of fungal superinfections is unknown^13^. *C. albicans*, a polymorphic commensal fungus, is immunocompetent and unaffected by the human microbiota. However, when disrupted, it can cause superficial skin and mucous membrane infections, leading to systemic infections^14^.

Glucans and -mannans recognize surface pattern recognition receptors on monocytes and macrophages, triggering an adequate immune response against *C. albicans*, including TLR2/4, NOD-like receptors, and C-type lectin receptors^15^. NLRP3 inflammasome activates receptors, producing proinflammatory cytokines such as TNF, IL-8, IL-17, IL-33, and IL-18 with fungicidal effects^16^. Moser et al.^17^ found that monocyte activation markers and cytokines IL-6, IL-8, TNF, IL-10, and sIL2R were enhanced in COVID-19 patients with *C. albicans* infection. This suggests that these patients may be more susceptible to *C. albicans* infection.

IL-33 triggers an innate defensive response after systemic CTC infection, increasing neutrophil phagocytosis and NK cell production of GM-CSF. It induces IL-17, which enhances protection against fungi and bacteria by recruiting neutrophils, synthesis of antimicrobial peptides, and barrier function^18^. Leukocytes struggle to phagocytize biofilm-associated cells, potentially causing COVID-19 patients to continue having *Candida* spp. infections during a cytokine storm, leading to increased immune cell count^19^. CTC lacks greater phagocytosis and lower fungal multiplication due to reduced T-cell count and ineffective phagocytosis, potentially due to T-cell reduction^20^. Severe COVID-19 increases secondary fungal infections, worsening clinical course in ARDS patients with pulmonary aspergillosis and candidiasis^21^.

Chen et al.^22^ discovered that five fungal infections were found in 99 COVID-19 patients at admission, including *Aspergillus flavus, Candida glabrata*, and *C. albicans*. Yang et al.^23^ discovered that fungal co-infections were found in 52 critically ill patients (3/52, 5.8%), including *A. flavus, A. fumigatus*, and *C. albicans*, according to the research.

Our findings provide initial evidence showing a weakened immune response to *C. albicans* and COVID-19 in Iraq, highlighting the need for attention and a good overview of the current understanding of COVID-19 and *C. albicans* co-infection. It also highlights the importance of further research in this area.

## CONCLUSION

Thrush candidiasis, an opportunistic fungal illness, is becoming more common as COVID-19. COVID-19 and *C. albicans* co-infection is associated with increased levels of proinflammatory interleukins and immune cells. Concurrent infection with COVID-19 and *C. albicans* (CTC) further increases these markers. There is a positive relationship between humoral and cellular immunity in patients with CTC. COVID-19 and CTC can lead to a hyperinflammatory state, which can increase the risk of complications and death. Patients with CTC may be more susceptible to other infections, including bacterial infections. Both humoral and cellular immunity are important for protecting against COVID-19 and *C. albicans* infection.
